# Reading Performances in Highly Myopic Patients and Correlation with the Topography of Atrophic Maculopathy

**DOI:** 10.1016/j.xops.2025.100743

**Published:** 2025-02-19

**Authors:** Matteo Mario Carlà, Carlos Mateo

**Affiliations:** 1 Ophthalmology Department, “Fondazione Policlinico Universitario A. Gemelli, IRCCS,” Rome, Italy; 2 Ophthalmology Department, Catholic University “Sacro Cuore,” Rome, Italy; 3 Vitreoretinal Surgery Department, Instituto de Microcirugía Ocular (IMO), Barcelona, Spain

**Keywords:** High myopia, Myopic atrophic maculopathy, Reading acuity, Reading performance, Reading speed

## Abstract

**Purpose:**

To evaluate how the topography of atrophic patches influences monocular and binocular reading performances in eyes with pathologic myopia.

**Design:**

Prospective single-center observational investigation.

**Participants:**

Sixty-two patients (112 eyes) affected by pathologic myopia (axial length [AXL] >26.5 mm). Only college graduates aged <65 years were selected.

**Methods:**

All patients underwent monocular and binocular reading evaluation using Colenbrander Reading Charts, taking into account the reading time and missed words/errors. Moreover, eyes underwent fundus photography and autofluorescence: the presence of chorioretinal atrophy within the central, 4 inner, and 4 outer ETDRS grid subfields was reviewed.

**Main Outcome Measures:**

Reading acuity (logarithm of the reading acuity determination [logRAD]); reading speed (words per minute [wpm]); percentage of errors/missed word; correlation with ETDRS subfield atrophy localization.

**Results:**

Mean AXL was 31.45 ± 2.21 mm. Monocularly, mean reading acuity was 0.37 ± 0.35 logRAD with an 8% ± 11% rate of missed or wrong words, whereas reading speed was 71.5 ± 27.8 wpm (range 25–125 wpm). Binocularly, mean reading acuity was 0.16 ± 0.16 logRAD with 5% ± 7% of missed or wrong words, whereas reading speed was 88.2 ± 18.0 wpm. Reading acuity was significantly associated with the presence of chorioretinal atrophy in the foveal central circle in univariate and multivariate analysis (*P* = 0.002). Conversely, reading speed negatively correlated with inner right subfield involvement in multivariate analysis (*P* = 0.008). Binocularly, reading acuity was associated with the presence of bilateral central atrophy (*P* = 0.001), whereas reading speed was associated with the presence of chorioretinal atrophy in the inner subfields on the horizontal plane in both eyes: bilateral inner right (*P* = 0.007) or inner left (*P* = 0.014) subfields; inner left OD (right eye)–inner right OS (left eye) (*P* = 0.002); inner right OD–inner left OS (*P* = 0.004).

**Conclusions:**

In highly myopic eyes, we reported a significant relationship between the topography of patchy chorioretinal atrophy and reading performance.

**Financial Disclosure(s):**

The author(s) have no proprietary or commercial interest in any materials discussed in this article.

High myopia, defined as an axial length (AXL) of >26.5 mm, is becoming more common in many nations, especially in Southeast Asia.^1^^,^^2^ After projections, nearly half of the global population will be affected by myopia by 2050, of whom 10% will suffer from high myopia.^3^ The pathologic elongation of the posterior sclera determines the development of degenerative changes, including posterior staphyloma, lacquer cracks, myopic traction maculopathy (MTM), chorioretinal atrophy (myopic atrophic maculopathy [MAM]), and secondary myopic neovascular maculopathy.^4^, ^5^, ^6^

These pathologic changes lead to variable degrees of visual degradation. In particular, several studies demonstrated the impact of pathologic myopia on near vision tasks, because reading skills are essential as early as school-age children, significantly affecting patients' quality of life.^7^^,^^8^ In recent years, the ability to study high myopic eyes has greatly improved thanks to the advancements in imaging technology.^9^^,^^10^ The use of OCT and fundus photography highlighted that the natural history of pathologic myopia follows a particular pattern of progression, with tessellated fundus giving way to widespread chorioretinal atrophy followed by patchy atrophy.^11^ Moreover, it has been shown that advanced maculopathy often advances even more quickly than the earlier stages.^11^

The assessment of best-corrected visual acuity in this condition still remains the gold standard for evaluating changes in visual function and ultimately directing therapy, as it happens in age-related macular degeneration (AMD).^12^^,^^13^ However, the near vision performances in the presence of macular disease have gained momentum in the last years: much more importance has been given to the effect of reduced contrast sensitivity,^14^ the crowding phenomenon,^15^ the visual span profile^16^ and fixation stability, and their impact on reading ability.^17^

Previous research has shown that monocular (and binocular) reading speed decreases with time and that there is a linear correlation between monocular reading speed and geographic atrophy (GA).^18^, ^19^, ^20^ Moreover, the topography of retinal pigment epithelium atrophy secondary to neovascular AMD (nAMD) was recently associated with changes in reading performances.^21^

To our knowledge, no thorough structure–function connection has been documented in highly myopic eyes with MAM. Therefore, the aim of this prospective research was to evaluate how the topography of myopic patchy atrophy influences monocular and binocular reading performances in high myopic eyes.

## Methods

This is a prospective single-center observational investigation on 112 high myopic eyes of 62 consecutive patients who visited the Instituto de Microcirugia Ocular, Barcelona, Spain, between January 2024 and April 2024. This research adhered to the tenets of the Declaration of Helsinki and was approved by the local Ethical Committee. All included subjects signed an informed consent.

The main inclusion criteria were the presence of pathologic myopia, defined as an AXL >26.50 mm or refractive myopia >8 D (diopters). Moreover, to remove the cultural bias and the possible confounding factor of age affecting reading speed, we selected only patients who completed college with an age of <65 years. Exclusion criteria were as follows: any kind of concomitant MTM (myopic retinoschisis, lamellar hole, macular hole, macular detachment) or previous surgical treatment for MTM, concomitant or previously treated myopic neovascular maculopathy, amblyopia, optic nerve disease (glaucoma, optic neuropathy), active uveitis, visually impacting cataract, corneal pathology affecting visual acuity (e.g., keratoconus) and severe systemic disease affecting ocular health.

At baseline, ophthalmic medical history was collected, along with a complete ophthalmic examination. Examinations included corrected distance visual acuity (CDVA), collected as Snellen equivalent and then converted to logarithm of the minimum angle of resolution (logMAR), reading performance assessment (explained below), and intraocular pressure evaluation. After pupil dilation, we performed spectral domain-OCT evaluation (Cirrus 5000 high-definition OCT, Carl Zeiss), ultra-widefield fundus photography, and blue fundus autofluorescence (FAF, Optomap, Optos). Axial length was also measured at the baseline (IOLMaster, Carl Zeiss Meditec).

### Reading Performance Assessment

Reading performance was assessed monocularly and binocularly, before pupil dilation, using the Spanish version of the Colenbrander Continuous Reading Charts (Precision Vision) with standard contrast, which includes 25 sentences with standardized grammatical construction, lexical difficulty, and syntactical complexity.^22^

The letter sizes, in Times Roman font, increase geometrically from 0.32 M to 6.3 M, or from 1.3 to −0.1 logarithm of the reading acuity determination (logRAD) (or 20/320 to 20/16 Snellen). The cards have a 40-cm string attached to them to help the patient maintain the proper reading distance, and they also include a ruler to help read at lower acuity levels at closer ranges. The test sentences vary in word count, as visible in Table 1, ranging from 6 to 9, but always include 42 characters total, including spaces. Each print size displays 1 phrase for decimal acuities between 0.063 and 0.1 and 2 sentences for those <0.12.

**Table 1:** Characteristics of the Colenbrander Continuous Reading Charts Used to Evaluate Reading Performance of This Cohort

Near Vision Line	Numbers of Words	Number of Sentences	Number of Characters per Sentence
20/320	7	1	42
20/250	7	1	42
20/200	7	1	42
20/160	17	2	42
20/125	17	2	42
20/100	16	2	42
20/80	16	2	42
20/63	15	2	42
20/50	15	2	42
20/40	16	2	42
20/32	17	2	42
20/25	16	2	42
20/20	13	2	42
20/16	14	2	42

Before the test, the refraction measure of patients who wore glasses was obtained using a lensometer. If not, the optimal refractive correction was determined by either retinoscopy or autorefractometer on the patients. Successively, to get the best correction, traditional subjective refraction was performed and all patients underwent successive reading tasks wearing trial lenses, to maximize the repeatability of the results. The room conditions, background lighting (luminance 120 cd/m^2^), reading distance (40 cm), and reading posture were kept constant for every assessment throughout the examination.

Patients were instructed to read the words out loud on the continuous reading charts as quickly as they could while being examined. Additionally, subjects were instructed to read until they were unable to do so, even in case of errors (such as mispronounced words or misread syllables) or missed words. Two independent examiners kept track of the number of errors or missed words and of the reading time of the entire lines of the chart, using a chronographer, and a mean of the 2 measurements was taken as valid.

After the examination, the following features were calculated: (1) maximum reading speed, determined by taking the fastest time across all print sizes and is expressed in words per minute (wpm); (2) reading acuity, defined as the logRAD at the smallest sentence that the patient was able to read in less than 30 seconds (range = −0.1 to 1.3 logRAD); and (3) percentage of errors or missed word reading of the smallest sentence that the patient was able to read. These features were evaluated both monocularly or binocularly.

### Imaging Protocol

**Figure 1:**
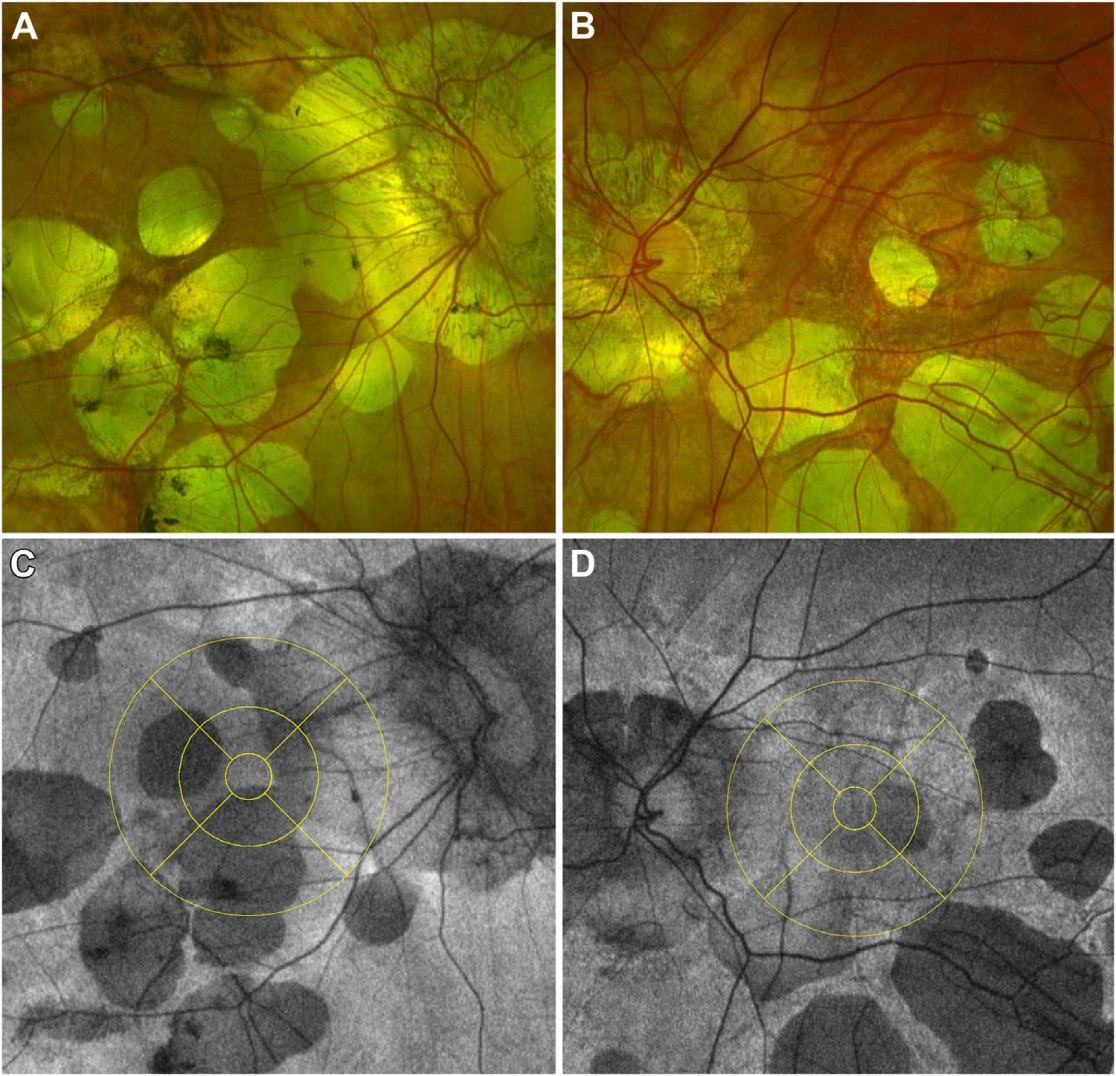
Fundus photography (**A, B**) and FAF (**C, D**) of 2 included eyes with patchy atrophic maculopathy. In the fundus autofluorescence image, the superimposed ETDRS grid was centered on the fovea and divided the macular area into 9 subfields.

After the reading performance evaluation, the patients underwent ultrawide fundus photography combined with FAF evaluation (Optomap, Optos). Subsequently, the ultrawide fundus and FAF images were cut to include only the 30° × 30° fovea-centered area. The coacquired infrared reflectance image derived from the OCT was registered to the FAF images using ImageJ software (National Institutes of Health) with vascular bifurcations as registration landmarks. After pixel-to-millimeter conversion, taking into account the known horizontal and vertical distance of the Macula Cross OCT scan (6 mm), the ETDRS grid was then superimposed to the FAF images and centered to the fovea using a preinstalled macro (Fig 1). In line with previous research,^21^ we defined the ETDRS subfield using the right–left localization, rather than the classic temporal–nasal: specifically, left subfields localized in the temporal and nasal areas in the right and left eyes, respectively. Conversely, right subfields localize in the nasal and temporal macular areas in the right and left eyes, respectively.

A sector was considered to be impacted by MAM if a zone of hypo-autofluorescence, indicating chorioretinal atrophy, >250 μm, resided inside it. All parameters were measured independently by 2 human graders.

### Statistical Analysis

Statistical analysis was performed using IBM SPSS software, version 27.0 (SPSS Inc). The Shapiro–Wilk test was employed to assess the normality of the sample. Quantitative variables were reported as mean and SD. The Pearson coefficient was employed for correlation analysis. Contributory factors influencing reading performance were analyzed using a univariate linear regression, and reading acuity and speed were considered as dependent variables. Subsequently, a multivariate model was developed to assess the impact of chorioretinal atrophy in the 9 ETDRS subfields on reading performances. The analysis was conducted both monocularly and binocularly. A *P* < 0.05 was deemed statistically significant.

## Results

Sixty-two Hispanic patients were enrolled in this study, for a total of 112 monocular evaluations. Fifty patients underwent also binocular evaluation: 12 patients were excluded from this kind of analysis because they had 1 eye affected by significant subfoveal atrophy, resulting in the inability to read even the biggest sentence (6.3 M). In these cases, only the better-seeing eye was included in the monocular analysis. Table 2 summarizes the demographics and clinical characteristics of the study cohort.

**Table 2:** Baseline Demographic and Clinical Characteristics of the Study Population

Characteristics	Value
Mean age, yrs	46.3 ± 8.4
Number of patients, n	62
Number of eyes, n	112
Eye laterality, OD/OS	58/54
Gender, Male/Female	19/43
CDVA, logMAR	0.44 ± 0.38
AXL, mm	31.45 ± 2.21
Reading performances: Monocular	
Reading acuity (logRAD)	0.37 ± 0.35
Reading speed (wpm)	71.5 ± 27.8
Missed/wrong words (%)	8 ± 11
Reading performances: Binocular	
Reading acuity (logRAD)	0.16 ± 0.16
Reading speed (wpm)	88.2 ± 18.0
Missed/wrong words (%)	5 ± 7

Data are shown as mean ± SD.

AXL = axial length; CDVA = corrected distance visual acuity; logMAR = logarithm of the minimum angle of resolution; logRAD = logarithm of the reading acuity determination; OD = right eye; OS = left eye; wpm = words per minute.

On average, AXL was 31.45 ± 2.21 mm. Monocular CDVA was 0.44 ± 0.38 logMAR (0.48 ± 0.28 Snellen equivalent). Monocular mean reading acuity was 0.37 ± 0.35 logRAD (0.54 ± 0.29 Snellen equivalent) with an 8% ± 11% rate of missed or wrong words, whereas mean reading speed was 71.5 ± 27.8 wpm (range 25–125 wpm). Binocularly, mean reading acuity was 0.16 ± 0.16 logRAD with 5% ± 7% of missed or wrong words, whereas reading speed increased to 88.2 ± 18.0 wpm on average.

**Figure 2:**
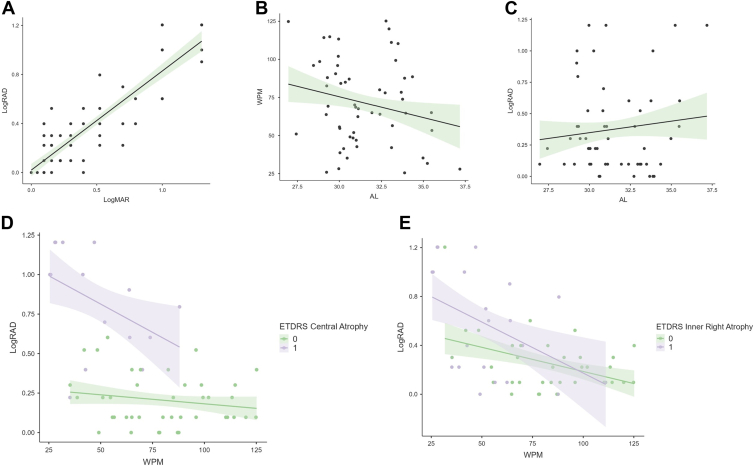
Scatter plots highlighting the correlations between several study parameters. Graphs report the line of regression, surrounded by a shadowed area indicating the standard error range. **A,** Corrected distance visual acuity (CDVA, logMAR) and reading acuity (logarithm of the reading acuity determination [logRAD]) showed a strong linear correlation (*r* = 0.64, 95% CI 0.56 to 0.72, *P* = 0.001). On the other hand, the correlation between axial length (AL) and both reading speed (**B,** words per minute [wpm]) and reading acuity (**C,** logRAD) was significant although not strong (*r* = −0.22, 95% CI −0.39 to −0.03, *P* = 0.02 and *r* = 0.21, 95% CI 0.02–0.38, *P* = 0.03, respectively). Based on the involvement of the central subfield of ETDRS grid **D,** eyes with central atrophy had significantly worse reading acuity (logRAD) and reading speed (wpm), whereas the involvement of the inner right subfield **E,** had a significant impact on reading speed (wpm) but not on reading acuity (logRAD). logMAR = logarithm of the minimum angle of resolution.

Reading acuity showed a significant but not strong correlation with AXL (Pearson’s *r* = 0.21, 95% confidence interval [CI] 0.02–0.38, *P* = 0.03), whereas a strong correlation was found with CDVA (r = 0.64, 95% CI 0.56–0.72, *P* = 0.001). On the other hand, reading speed correlated with AXL (*r* = −0.22, 95% CI −0.39 to −0.03, *P* = 0.02) but not with CDVA (*r* = 0.12, 95% CI −0.06 to 0.30, *P* = 0.28). Moreover, the rate of missed or wrong words correlated with both reading acuity (*r* = 0.31, 95% CI 0.18–0.42, *P* = 0.01) and reading speed (Pearson’s *r* = −0.55, 95% CI −0.66 to −0.41, *P* = 0.001). Between male and female subjects, no differences were reported in terms of CDVA (*P* = 0.45), reading acuity (*P* = 0.78), or reading speed (*P* = 0.61). Scatter plots showing the correlation between the analyzed variables are reported in Figure 2.

**Figure 3:**
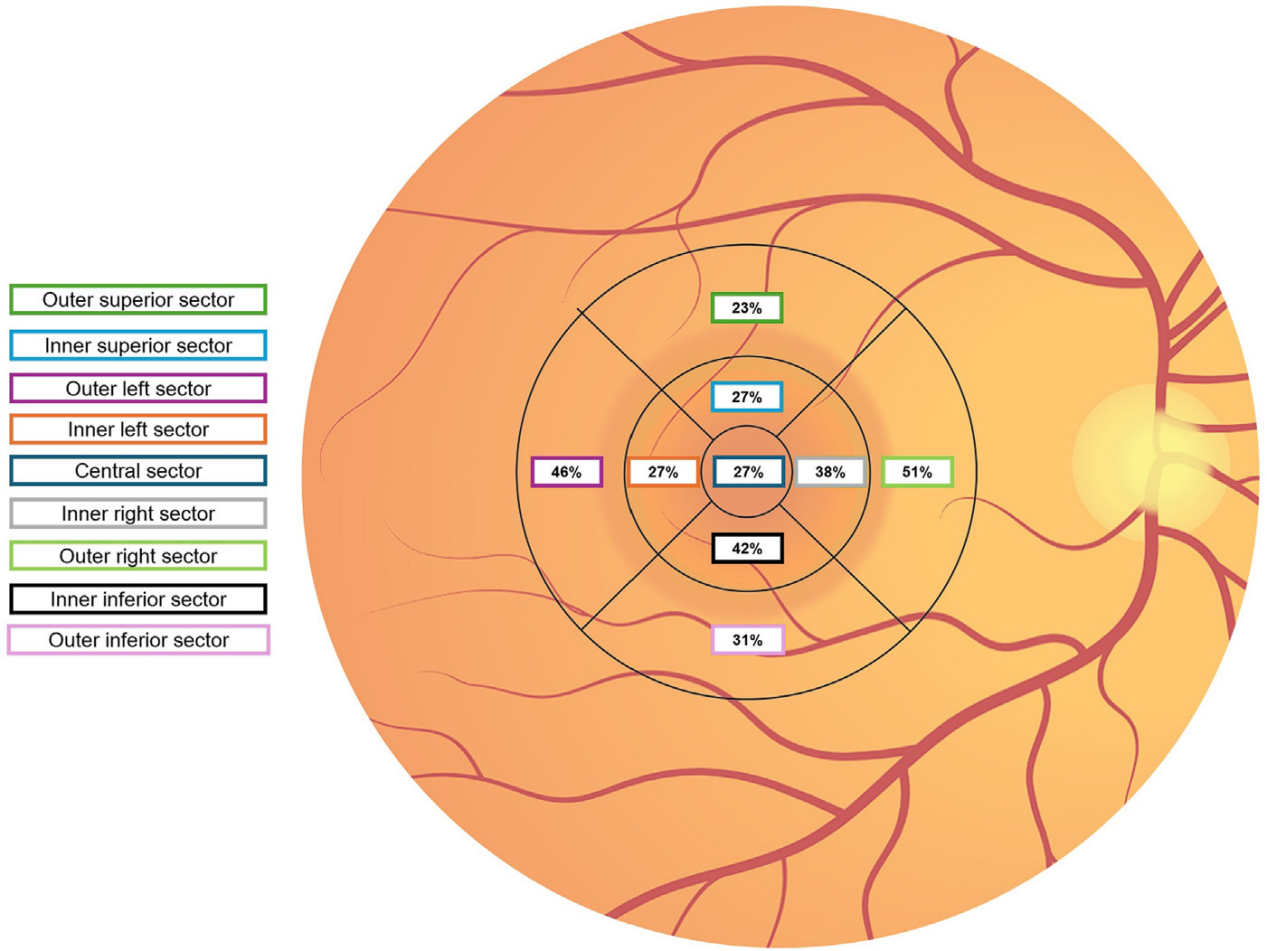
Prevalence (%) of the ETDRS grid constitutes the prevalence (%) of retinal pigment epithelium atrophy in each subfield. Overall, 96 of 110 eyes (87%) showed chorioretinal atrophy in the macular area, majorly involving the inner inferior, outer left, and outer right subfields.

Overall, 96 of 112 eyes (86%) showed any kind of chorioretinal atrophy in the ETDRS grid area, primarily located at the inner inferior (42%), outer left (56%), and outer right (51%) subfields. The prevalence of chorioretinal atrophy split in ETDRS subfields is displayed in Figure 3.

### Monocular Linear Regression Analyses

The summary of univariate and multivariate regression analyses defining reading acuity and reading speed as dependent variables is visible in Table 3. In univariate regression analysis, reading acuity was significantly associated with the presence of chorioretinal atrophy in the foveal central circle (*P* < 0.001), and this association was confirmed in multivariate analysis (*P* = 0.002). Conversely, reading speed was negatively associated with the presence of atrophy in the central (*P* = 0.031), outer inferior (*P* = 0.037), inner left (*P* = 0.048), and inner right (*P* = 0.005) subfields in univariate analysis. However, only inner right subfield involvement kept a significant correlation in multivariate analysis (*P* = 0.008) (Fig 2).

**Table 3:** Predictors of Reading Performances in Univariate and Multivariate Analysis

Predictors	Reading Acuity				Reading Speed			
	Univariate		Multivariate		Univariate		Multivariate	
	95% CI	*P*	95% CI	*P*	95% CI	*P*	95% CI	*P*
Age (yrs)	−0.02 to +0.01	0.389	—	—	−0.36 to +0.66	0.549	—	—
Axial length (mm)	−0.50 to +0.01	0.032	—	—	−4.05 to +0.24	0.081	—	—
CDVA (logMAR)	+0.50 to +0.76	<0.001	—	—	−27.10 to +8.44	0.300	—	—
Sex (Male/Female)	−0.14 to +0.03	0.198	—	—	−11.21 to +12.03	0.944	—	—
ETDRS central	+0.55 to +0.82	<0.001	+0.16 to +0.41	0.002	−35.07 to −0.05	0.031	−28.34 to +1.72	0.101
ETDRS inner superior	−0.34 to −0.02	0.027	−0.18 to +0.06	0.340	−4.42 to +28.60	0.149	−2.28 to +14.12	0.225
ETDRS outer superior	−0.13 to +0.09	0.722	−0.21 to +0.08	0.388	−12.58 to +17.24	0.757	−13.31 to +16.58	0.828
ETDRS inner inferior	−0.14 to +0.03	0.224	−0.10 to +0.14	0.732	−20.66 to +3.28	0.153	−19.66 to +3.52	0.170
ETDRS outer inferior	−0.02 to +0.16	0.115	–0.01 to +0.24	0.063	+0.83 to +25.18	0.037	−1.29 to +14.11	0.155
ETDRS inner left	−0.11 to +0.06	0.620	−0.16 to +0.08	0.500	−20.53 to −3.18	0.048	−21.70 to +1.89	0.099
ETDRS outer left	−0.12 to +0.03	0.224	−0.06 to +0.14	0.396	−18.01 to +2.48	0.136	−11.65 to +4.14	0.217
ETDRS inner right	−0.12 to +0.06	0.522	−0.12 to +0.13	0.931	−30.76 to −5.77	0.005	−30.99 to −5.84	0.008
ETDRS outer right	−0.13 to +0.02	0.168	−0.09 to +0.11	0.826	−13.48 to +6.91	0.524	−16.98 to +2.64	0.150

95% CI and *P* values of excluded cases in the multivariable analysis were not reported.

CI = confidence interval; CDVA = corrected distance visual acuity; logMAR = logarithm of the minimum angle of resolution.

### Binocular Linear Regression Analysis

In 50 patients, multivariate analysis was performed to evaluate the correlation between binocular reading acuity and speed with the topography of chorioretinal atrophy, specifically focusing on the combination of atrophy in the inner circle of the 2 eyes (Table 4).

**Table 4:** Multivariate Analysis of Binocular Reading Performances (n = 50 Patients) with the Topography of Myopic Maculopathy in Both Eyes, Focusing on the Inner Ring of the ETDRS Chart (Central, Inner Superior, Inner Inferior, Inner Left, Inner Right)

Predictor	Reading Acuity		Reading Speed (Wpm)	
	Coefficient	*P*	Coefficient	*P*
OD central	0.18	0.018	−14.61	0 .031
OD inner superior	0.19	0.216	−4.60	0 .201
OD inner inferior	−0.01	0.885	−19.23	0.147
OD inner left	−0.004	0.432	−20.08	0.062
OD inner right	0.13	0.013	−26.00	0.002
OS central	0.25	0.001	−16.98	0.040
OS inner superior	−0.19	0.416	4.43	0.572
OS inner inferior	0.03	0.606	−14.47	0.156
OS inner left	0.04	0.438	13.25	0.159
OS Inner right	0.17	0.009	−10.79	0.072
OD central ×OS central	0.50	0.001	−23.03	0.009
OD central × OS inner superior	0.29	0.039	−10.62	0.151
OD central × OS inner inferior	−0.21	0.126	8.18	0 .081
OD central × OS inner left	0.24	0.012	−34.42	0.044
OD central × OS inner right	0.47	0.002	−71.62	0.001
OD inner superior × OS central	−0.25	0.108	11.16	0.561
OD inner superior × OS inner superior	0.09	0.416	1.14	0.948
OD inner superior × OS inner inferior	−0.26	0.065	−8.10	0.102
OD inner superior × OS inner left	0.09	0.648	−20.51	0.014
OD inner superior × OS inner right	−0.19	0.283	−46.83	0 .041
OD inner inferior × OS central	−0.18	0.151	19.53	0.240
OD inner inferior × OS inner superior	0.31	0.141	11.76	0.541
OD inner inferior × OS inner inferior	0.02	0.676	−2.91	0.698
OD inner inferior × OS inner left	−0.01	0.934	−42.05	0.014
OD inner inferior × OS inner right	−0.11	0.313	10.47	0.469
OD inner left × OS central	0.13	0.206	−22.11	0.029
OD inner left × OS inner superior	0.17	0.209	−9.71	0.575
OD inner left × OS inner inferior	−0.23	0.017	17.24	0.129
OD inner left × OS inner left	0.01	0.947	−20.55	0.014
OD inner left × OS inner right	0.28	0.058	−52.16	0.002
OD inner right × OS central	−0.71	<0.001	−11.05	0.028
OD inner right × OS inner superior	−0.19	0.101	27.00	0.152
OD inner right × OS inner inferior	0.11	0.247	−22.62	0.206
OD inner right × OS inner left	0.16	0.141	−38.82	0.004
OD inner right × OS inner right	−0.20	0.037	−49.73	0.007

OD = right eye; OS = left eye; Wpm = words per minute.

Reading acuity was significantly associated with the presence of chorioretinal atrophy in the central subfield of both eyes (*P* = 0.001) or in the central subfield of 1 eye and the left or right inner subfield of the fellow eye (e.g., central subfield OD (right eye)–inner left OS (left eye) [*P* = 0.012], inner right OD–central subfield OS [*P* < 0.001]). In a similar way, reading speed was associated with bilateral central subfield atrophy (*P* = 0.009) but also with the presence of chorioretinal atrophy in the inner subfields on the horizontal plane in both eyes: bilateral inner right (*P* = 0.007) or inner left (*P* = 0.014) subfields; inner left OD–inner right OS (*P* = 0.002); inner right OD–inner left OS (*P* = 0.004).

## Discussion

Several investigations highlighted that myopic eyes perform less efficiently than emmetropic eyes in terms of CDVA and contrast sensitivity, with major declines as myopia increases.^23^, ^24^, ^25^ In this research, we measured the reading performances of highly myopic eyes and sought a correlation between the topography of MAM lesions and reading impairment.

The natural history of degenerative myopia has been thoroughly investigated, highlighting a typical pattern of progression ranging from a tessellated fundus, patchy chorioretinal atrophy, and foveal atrophy.^26^, ^27^, ^28^ In our prospective research, we identified a correlation between the location of atrophy patches, identified by fundus photography and FAF, with both maximum reading acuity and reading speed. Specifically, as expected, the main factor influencing both monocular and binocular reading acuity was the involvement of the central ETDRS subsector, suggesting subfoveal atrophy. On the other hand, reading speed in monocular examination strongly correlated with the presence of chorioretinal atrophy in the inner right ETDRS subfield. Binocularly, the presence of patchy atrophy involving the horizontal foveal and perifoveal plane (e.g., inner left, central, and inner right ETDRS subfield) was significantly associated with lower reading speed.

The correlation between structural damage and impaired reading ability was recently explored in AMD patients.^21^^,^^29^, ^30^, ^31^ In a study including 85 participants (150 eyes), Kunzel et al^29^ prospectively examined the link between the metrics derived from a different logarithmic near vision scale, the Radner reading charts, and AMD-associated GA. In a successive investigation, Aslam et al^31^ showed that reading performances are associated with structural biomarkers reflecting retinal pigment epithelium atrophy. Similarly, Hoerster et al^32^ reported that reading performance sensitivity may predict exudation recurrences in nAMD patients receiving anti-VEGF injections. Out of the 40 patients whose structural OCT revealed a recurrence, 7.5% reported increased metamorphopsia or scotoma, and 10% reported worse reading skills.^32^ More recently, Ricardi et al^21^ pointed out a substantial correlation between reading performances, measured with the Radner Chart, and the presence and location of macular atrophy identified by structural OCT, in nAMD patients previously treated with anti-VEGF.

In line with previous reports, we demonstrated a similar correlation between structural and functional reading abilities in patients with myopic maculopathy. In our research, the evaluation of reading performance was carried out with Colenbrander Continuous Reading Charts, a previously validated logarithmic chart, which showed a similar coefficient of variability in terms of reading acuity when compared with other equivalent charts, such as the Radner and MNRead Charts.^22^ We reported lower reading speed values (71.5 wpm on average) when compared with patients with nAMD from the investigation conducted by Ricardi et al^21^ (144 wpm on average), but our results were comparable to those reported by Kunzel et al^29^ in GA eyes (52.8 wpm on average),^33^ probably justified by the presence of larger areas of chorioretinal or retinal pigment epithelium atrophy in myopic maculopathy and GA.

Similar to previous studies, maximum reading acuity was significantly jeopardized by the presence of MAM lesions within the central ETDRS subfield, both monocularly and binocularly, evaluated with fundus photography and FAF. In fact, in the presence of a central chorioretinal atrophy, as already studied in patients with AMD, the visual span profile may have an irregular form, as letter recognition accuracy may significantly decline at letter locations corresponding to the central scotoma.^16^ Moreover, in myopic eyes, higher AXL values and worse CDVA were associated with worse reading acuity in the univariate analysis. In particular, in our cohort, we highlighted a strong linear correlation between CDVA and reading acuity (*r* = 0.64). Conversely, previous reports regarding nAMD showed a disproportionate worsening of near compared with far vision,^34^ highlighting that reading acuity was generally much more impacted than CDVA.^21^ Nevertheless, our results are similar to those reported by Kunzel et al^29^ in patients with GA, in which CDVA strongly correlated with reading acuity (*r* = 0.82) and was the variable with the greatest influence on reading performances. These findings suggest that the involvement of the parafoveal and perifoveal areas, more likely to happen in GA and MAM rather than nAMD, may cause proportional impairment of near and far visual acuity, even if literature is still controversial on this subject.^35^

Consistent with the results on nAMD and GA patients, the presence of chorioretinal atrophy in the inner right ETDRS subfield strongly correlated with lower maximal reading speeds.^21^^,^^29^ As demonstrated before, reading speed seems to provide more insight into retinal function near the fovea: because our patients read texts with left-to-right directionality, this specific subfield may impact the natural transitioning from one word to the next in a phrase. As a result, a decline in retinal function in this sector may hinder the natural flow of reading and eventually result in a slower reading speed. Moreover, when performing binocular evaluation, we highlighted that the bilateral presence of chorioretinal atrophy in the inner subfields on the horizontal plane (central, right, and left ETDRS subfields) was associated with lower reading speed values, suggesting that the integration of visual stimuli derived from these sectors is crucial for reading fluency. It was already demonstrated that the crowding phenomenon, such as the degradation of letter recognition when the letter is surrounded closely by other letters, is a major bottleneck for reading speed in patients with macular disease.^15^^,^^36^ Moreover, the magnitude and extent of crowding are yet larger in the normal periphery than at the fovea.^37^ Based on this assumption, we hypothesize that the combination of parafoveal atrophy and the stretching of retinal structural components typical of myopic eyes^38^ may further trigger crowding and undermine reading speed.

Of note, our study has several limitations. First, even if we conducted a prospective analysis, we focused on a small cohort and our result may lack generalizability. Second, even if we excluded eyes with MTM and myopic neovascular maculopathy, different staphyloma morphologies, optic nerve subclinical impairment, and choroidal defects may have a role in myopic degeneration, influencing reading performances. Moreover, the superimposition of the ETDRS grid on fundus photography of a myopic eye, even if performed after OCT-guided fovea centering, may not be perfectly precise due to the anomalous curvature of myopic staphyloma, overestimating or underestimating the dimensions of each ETDRS subfield. In addition, we did not use microperimetry to explore fixation patterns within our study group; thus, we could have missed the presence of extrafoveal preferred retinal locus or cases with fixation instability.

In conclusion, the current investigation pointed out that, similar to GA and nAMD patients, myopic patients also have a significant correlation between reading performances and retinal structural changes. Monocular and binocular reading acuity was primarily jeopardized in the case of central atrophy, whereas reading speed was significantly impacted by the presence of chorioretinal atrophic changes in the inner right subfield in monocular analysis, or in the horizontal plane binocularly. Because myopic changes are often progressive, the analysis of reading acuity and speed may offer a quantitative parameter of functional impairment in areas with patchy atrophy, or even acquire a predictive value.
